# Systemic Granulomatous Mycobacteriosis in Orbiculate Batfish (*Platax orbicularis*) Associated with *Mycobacterium marinum*-like Organism in an Aquarium in South Korea

**DOI:** 10.3390/vetsci13050489

**Published:** 2026-05-18

**Authors:** Chi Yong Kim, Young-Hyun Goo, Sukhun Oh, Sun-Hee Do

**Affiliations:** 1 Department of Companion Animal Health and Science, Silla University, Busan 46958, Republic of Korea; ckim@silla.ac.kr; 2 Department of Veterinary Clinical Pathology, College of Veterinary Medicine, Konkuk University, Seoul 05029, Republic of Korea; 3 OSH Veterinary Clinic, Seoul 04733, Republic of Korea

**Keywords:** *Mycobacterium marinum*, orbiculate batfish, *Platax orbicularis*, granuloma, aquarium fish, non-tuberculous mycobacteria

## Abstract

In this report, we describe systemic granulomatous mycobacteriosis in an orbiculate batfish from an aquarium in South Korea. Gross examination of the deceased fish showed multifocal nodular lesions in multiple internal organs including the gills, spleen, and kidney. Histopathological analysis demonstrated severe chronic systemic granulomatous inflammation, and Ziehl–Neelsen staining highlighted abundant intralesional acid-fast bacilli. Molecular analysis based on partial sequencing of the 16S ribosomal RNA (rRNA) and heat shock protein 65 (*hsp65*) genes showed that the detected organism was most closely related to *Mycobacterium marinum.* Because the molecular analysis was performed using partial sequences obtained from formalin-fixed, paraffin-embedded tissues, definitive species-level identification was not possible. This case represents systemic granulomatous mycobacteriosis associated with a *Mycobacterium marinum*-like organism in orbiculate batfish in an aquarium in South Korea and emphasizes the need for continuous disease surveillance and improved diagnostic awareness of non-tuberculous mycobacterial infections in ornamental and public aquarium fish.

## Simple Summary

This report describes systemic granulomatous disease in an orbiculate batfish from an aquarium in South Korea. Gross and histopathological examinations revealed severe multifocal granulomas containing acid-fast bacteria across multiple organs, including the gills, spleen, and kidney. Subsequent molecular analysis showed that the detected bacterium was interpreted as being most closely related to *Mycobacterium marinum*. These findings highlight the need for continued monitoring of bacterial infections in aquarium fish and support awareness of potential zoonotic risks to handlers and aquarists.

## 1. Introduction

*Mycobacterium marinum* is a slow-growing, non-tuberculous mycobacterium [1]. It belongs to the *Mycobacterium marinum* complex, which comprises closely related species such as *M. shottsii*, *M. pseudoshottsii*, and *M. ulcerans* [2]. It is an opportunistic pathogen found in both freshwater and marine environments, particularly in tropical and subtropical regions, and is capable of infecting a wide range of organisms as well as humans [3]. In aquatic environments, *M. marinum* has a broad host range that includes amphibians, reptiles, and various fish such as goldfish, zebrafish, salmon, and bass [4,5]. The pathological spectrum in fish ranges from localized lesions to systemic granulomatous diseases affecting internal organs, including the liver, spleen, kidney, and heart [6,7]. Histologically, granulomatous lesions in fish consist primarily of aggregates of epithelioid macrophages and peripheral lymphocytes, with variable central necrosis [8]. Infected fish may exhibit lethargy, anorexia, emaciation, and ulcerative skin lesions, although subclinical infections are also common [3,9]. Diagnosis is established using a combination of clinical findings, histopathology, bacterial culture, and molecular identification based on sequence analysis of the 16S ribosomal RNA (rRNA) and heat shock protein 65 (*hsp65*) genes [10].

In addition to its veterinary relevance, *M. marinum* is recognized as a zoonotic pathogen [11]. In humans, it is associated with fish tank granuloma and typically presents as localized cutaneous or subcutaneous lesions following exposure to infected fish or contaminated water [3]. Immunosuppressed individuals are at a greater risk for disseminated infection [3]. Therefore, *M. marinum* and related species pose a potential occupational hazard for aquaculture workers and fish handlers. Despite its significance, documented reports of systemic mycobacterial infections in public aquarium species remain limited. Orbiculate batfish are tropical marine fish commonly displayed because of their distinctive appearance and schooling behavior. To the best of our knowledge, systemic granulomatous mycobacteriosis associated with a *Mycobacterium marinum*-like organism has not previously been reported in orbiculate batfish in an aquarium in South Korea. This study describes such a case and highlights the importance of routine disease surveillance and biosecurity in managed aquatic systems.

## 2. Case Description

### 2.1. Gross Findings

**Figure 1:**
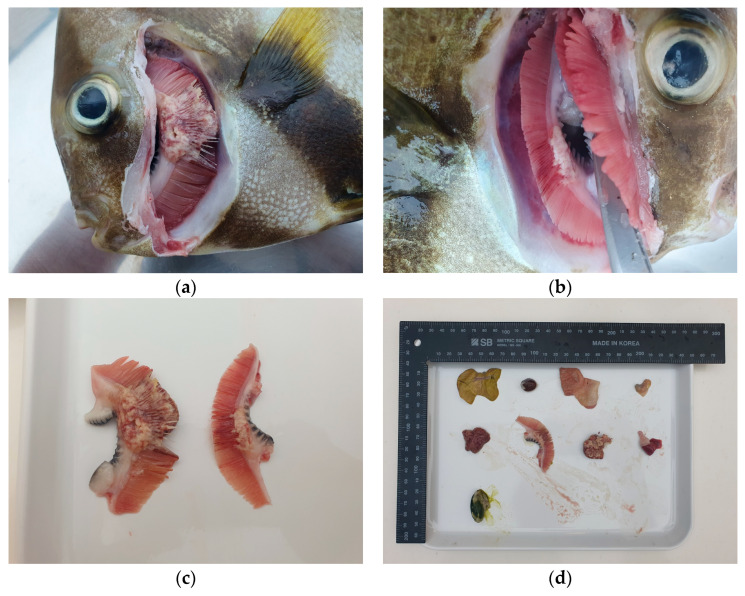
Gross lesions in an orbiculate batfish (*Platax orbicularis*). (**a**) Multifocal nodular lesions were observed on the left gill filaments after removal of the left operculum. (**b**) Right gill filaments, separated after a right opercular incision, revealing nodular lesions between the filaments. (**c**) Gross image of the left and right gill showing multifocal hemorrhage and pale nodular lesions. (**d**) Representative internal organs collected at necropsy for subsequent histopathological examination.

A 33-cm-long, 1.3 kg orbiculate batfish (*Platax orbicularis*) was found dead in an aquarium in South Korea and was subsequently submitted for necropsy. On gross examination, the gill filaments were diffusely swollen and distorted by multifocal to coalescing pale to whitish nodular lesions, as shown in Figure 1. These nodules were irregular in shape and variable in size, with some areas exhibiting partial coalescence, multifocal hemorrhage, and loss of normal filament architecture (Figure 1a,c). After opening the right operculum, similar nodular lesions were also observed along the gill (Figure 1b). Representative sections of gills and internal organs were collected, fixed in 10% neutral buffered formalin, and processed into formalin-fixed, paraffin-embedded (FFPE) tissues for histopathological evaluation (Figure 1d). Detailed husbandry and environmental records, including water quality, stocking density, filtration, and information on cohabiting species, were not available for retrospective review in this case.

### 2.2. Histopathological Findings

**Figure 2:**
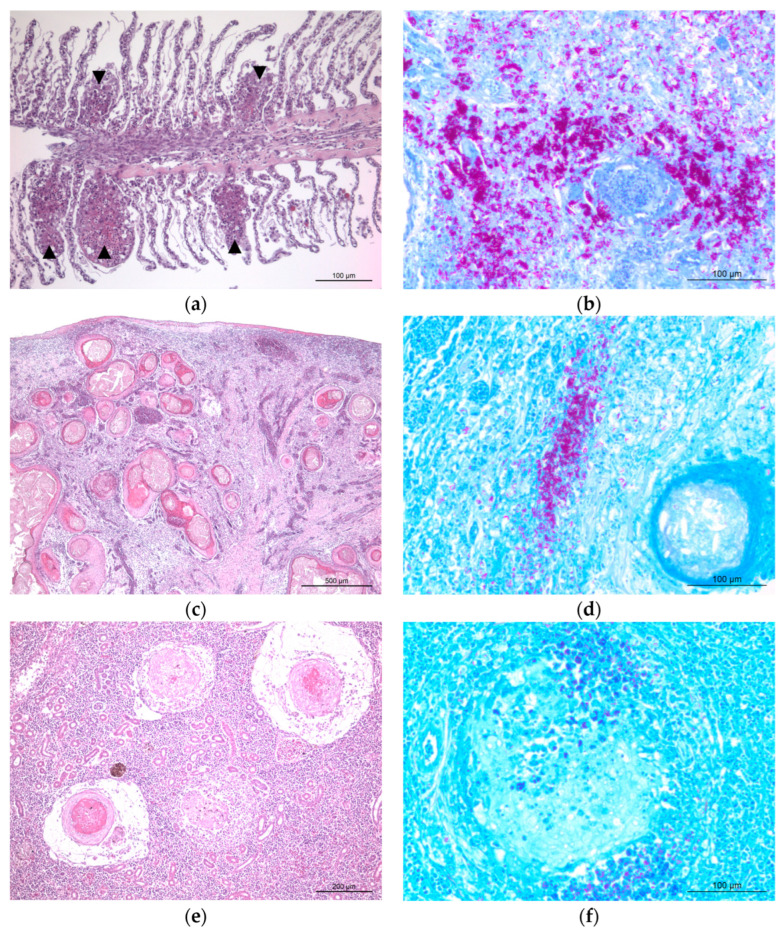
Representative histopathological lesions of systemic granulomatous mycobacteriosis in an orbiculate batfish. Scale bars as indicated. (**a**) H&E-stained section of the gill showing multifocal granulomatous inflammation (arrowheads). (**b**) Ziehl–Neelsen-stained section of the gill demonstrating numerous acid-fast bacilli within granulomatous lesions. (**c**) H&E-stained section of the spleen showing multifocal to coalescing granulomas with central necrotic cores. (**d**) Ziehl–Neelsen-stained section of the spleen highlighting abundant acid-fast bacilli within the necrotic core and surrounding inflammatory cells. (**e**) H&E-stained section of the kidney showing multifocal granulomatous lesions with central necrosis. (**f**) Ziehl–Neelsen-stained section of the kidney demonstrating acid-fast bacilli within granulomatous lesions.

For histopathological evaluation, FFPE tissue sections were stained with either hematoxylin and eosin (H&E) for routine morphological assessment and with Ziehl–Neelsen stain for the detection of acid-fast bacilli. In the gill, multifocal granulomatous lesions were observed, resulting in marked disruption of the normal filament architecture and branchial arch structure (Figure 2a). Ziehl–Neelsen-stained sections of the gill demonstrated abundant rod-shaped acid-fast bacilli within the granulomatous lesions (Figure 2b). The most severe lesions were identified in the parenchymal organs, particularly the spleen and kidney. In the spleen, the normal hematopoietic architecture was extensively replaced by multifocal to coalescing granulomas characterized by prominent central necrotic cores composed of eosinophilic cellular debris and surrounded by epithelioid macrophages (Figure 2c). Ziehl–Neelsen staining of the spleen highlighted abundant acid-fast bacilli within the necrotic cores and adjacent inflammatory cells (Figure 2d). Similar granulomatous lesions with central necrosis were observed in the kidney (Figure 2e), and Ziehl–Neelsen staining demonstrated numerous acid-fast bacilli within these lesions (Figure 2f). Overall, these findings were consistent with chronic systemic granulomatous inflammation associated with acid-fast bacilli.

### 2.3. Molecular Analysis

To further characterize the acid-fast bacteria observed in the histological sections, molecular analysis was performed. Although fresh tissue samples are generally preferred for optimal pathogen identification, only FFPE specimens were available in the present case. Therefore, the FFPE tissues originally prepared for histological examination were used for molecular identification. Despite the technical challenges of detecting mycobacterial DNA from FFPE specimens, primarily because of nucleic acid degradation and cross-linking induced by formalin fixation, DNA extraction and subsequent PCR amplifications were successfully achieved [12]. In brief, DNA was extracted from the FFPE tissue sections (10 µm thick) using the QIAamp DNA FFPE Advanced Kit (QIAGEN, Hilden Germany) according to the manufacturer’s instructions, and the final product was eluted in the elution buffer. To improve analytical sensitivity, a nested PCR strategy was used to target both 16S rRNA and *hsp65* genes. For the amplification of the *hsp65* gene, two primer sets were adopted from a previous study [13]. For the 16S rRNA, specific primer sets were designed to optimize amplification from the FFPE-derived templates. In the first round of PCR, the extracted DNA served as the initial template, and resulting primary PCR products were then used as templates for the second-round (nested) PCR. The sequences of all forward and reverse primers used for these reactions are listed in Table 1. A no-template negative control was included in each PCR run, and pre-PCR setup, amplification, and post-PCR handling were performed in physically separated work areas using aerosol-resistant filter tips to minimize contamination. Both rounds of PCR amplifications were performed under the following conditions: initial denaturation at 94 °C for 2 min, followed by 30 cycles of denaturation at 94 °C for 30 s, annealing at 50 °C for 30 s, and extension at 68 °C for 30 s, with a final extension at 72 °C for 5 min. Amplicons were confirmed by agarose gel electrophoresis and subjected to direct sequencing. The expected amplicon size of the second-round 16S rRNA product was 200 bp, whereas those of the first- and second-round PCR products were 463 bp and 439 bp, respectively.

Basic Local Alignment Search Tool (BLAST, https://blast.ncbi.nlm.nih.gov/Blast.cgi, accessed on 21 April 2026) analysis of partial 16S rRNA and *hsp65* gene sequences demonstrated that the detected organisms were most closely related to *M. marinum*, as summarized in Table 2. Specifically, the 16S rRNA sequences obtained from the gill and spleen exhibited 97.3 and 95.7% identity, respectively, to reference sequences of *M. marinum*. Furthermore, the *hsp65* sequences derived from the gill and kidney showed higher similarities, 99.5 and 100.0%, respectively.

**Figure 3:**
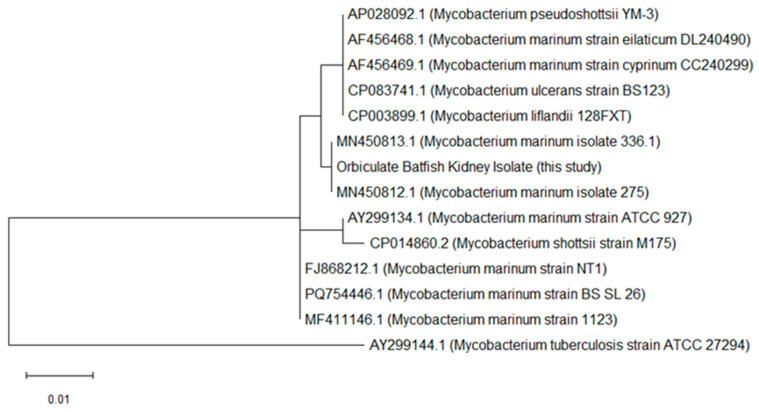
Phylogenetic tree based on partial *hsp65* gene sequence obtained from the kidney tissue of the orbiculate batfish in this study (GenBank accession number: PZ290492) and closely related *Mycobacterium* strains. A reference sequence of *M. tuberculosis* was additionally included to provide phylogenetic context for the tree interpretation. Sequence alignment and phylogenetic tree construction were performed using the Maximum Likelihood method with the Tamura-Nei model in MEGA version 12. Bootstrap analysis was performed with 1000 replicates. Positions containing gaps and missing data were eliminated using the complete deletion option. GenBank accession numbers and strain names are shown for all sequences included in the analysis. The scale bar indicates the number of nucleotide substitutions per site.

Phylogenetic analysis based on the partial *hsp65* gene sequences was performed using the Maximum Likelihood method and the Tamura-Nei model in MEGA version 12. Twelve reference sequences representing closely related taxa within the *M. marinum* complex and related fish-associated non-tuberculous mycobacteria were included, together with the highest-quality sequence obtained from the kidney tissue of the orbiculate batfish in this study. The sequences were aligned using MUSCLE, and the resulting phylogenetic tree demonstrated that the kidney-derived sequence clustered closely with reference strains of *M. marinum* (Figure 3). Specifically, it formed a tight clade with *M. marinum* isolates, 275 (MN450812.1) and 336.1 (MN450813.1), while remaining separated from other closely related mycobacterial species, including *M. pseudoshottsii*, *M. ulcerans*, and *M. shottsii*. Because the other sequences obtained in this study were shorter and of lower comparative value for tree construction, they were used for BLAST-based comparison rather than inclusion in the phylogenetic tree. These molecular findings, together with the histopathological evidence of acid-fast bacilli, support the presence of a systemic granulomatous disease associated with a pathogen closely related to *M. marinum*.

## 3. Discussion

This study describes a case of systemic granulomatous disease in an orbiculate batfish from an aquarium in South Korea, characterized by multifocal granulomas in multiple organs and abundant intralesional acid-fast bacilli. Histopathological examination revealed granulomatous lesions in the gills, spleen, and kidney, with the most severe changes observed in the parenchymal organs. These findings are consistent with previous reports of piscine mycobacteriosis, in which granulomas composed of epithelioid macrophages and associated inflammatory cells are commonly observed. The distribution of lesions across multiple organs supports the diagnosis of a systemic mycobacterial infection. Differential diagnoses for multifocal granulomatous lesions in fish include infections caused by other non-tuberculous mycobacteria, *Nocardia* spp., certain fungal agents, and chronic parasitic or foreign-body-associated granulomatous inflammation; however, the presence of abundant intralesional acid-fast bacilli strongly supported a mycobacterial etiology in the present case. Similar systemic granulomatous lesions associated with *Mycobacterium* spp. have been described in other ornamental and cultured fish species, particularly involving the spleen, kidney, and gills [14,15]. The lesion distribution and abundant intralesional acid-fast bacilli observed in the present case were broadly consistent with those previously reported in piscine mycobacteriosis [14,15]. However, unlike culture-confirmed cases, the present report relied on FFPE-based molecular analysis, which limited definitive species-level identification [12].

Molecular characterization based on the partial sequencing of the 16S rRNA and *hsp65* genes suggested that the detected organism was most closely related to *M. marinum.* However, definitive species-level identification remained challenging because the analysis was performed using DNA extracted from FFPE tissues, which can compromise DNA length and quality. In addition, the phylogenetic analysis was based on a single highest-quality *hsp65* sequence, and branch-support values were not displayed in the current tree presentation. Bacterial culture and isolation were also not available in this case, representing an important diagnostic limitation. Accordingly, the etiological agent is most appropriately and cautiously designated as an *M. marinum*-like organism rather than definitively identified as *M. marinum*. Because only FFPE specimens had been preserved for retrospective analysis, fresh tissues suitable for mycobacterial culture were not available in this case; therefore, culture-based confirmation could not be performed.

In aquatic environments, *M. marinum* and related species are widely distributed and are recognized as opportunistic pathogens infecting a broad range of marine and fresh-water fish. In the present case, the severe systemic granulomatous lesions observed in the orbiculate batfish were consistent with findings reported in previous studies across susceptible species. Because detailed tank history, water-quality data, stocking density, filtration records, quarantine information, and information on tank mates were unavailable in this case, environmental factors are discussed here only as general risk factors reported in aquarium mycobacteriosis.

In addition to its veterinary significance, *M. marinum* is a well-recognized zoonotic pathogen responsible for fish tank granuloma in humans. Accordingly, the presence of a *M. marinum*-like organism in aquarium fish warrants general awareness of potential occupational exposure among aquarists, fish handlers, and other personnel frequently exposed to aquatic environments. However, because no viable bacterial isolate, human exposure assessment, or human infection data were available in this case, the zoonotic implications should be interpreted cautiously. These findings highlight the importance of implementing suitable biosecurity measures and maintaining awareness of potential zoonotic risks in aquatic systems.

## 4. Conclusions

This report describes a case of systemic granulomatous disease in an orbiculate batfish from a public aquarium in South Korea. Gross and histopathological findings, together with molecular analysis based on partial 16S rRNA and *hsp65* gene sequences, indicated that the detected organism was most closely related to *Mycobacterium marinum*. Because definitive species-level identification was limited using partial FFPE-derived sequences and the lack of bacterial isolation, the etiologic agent is best interpreted as a *M. marinum*-like organism. This case highlights the importance of routine disease surveillance and improved diagnostic awareness of non-tuberculous mycobacterial infections in aquarium fish, as well as the need for appropriate biosecurity awareness regarding potential zoonotic risk.

## Figures and Tables

**Table 1:** Primer sets used for the nested PCR amplification of mycobacterial genes and expected amplicon sizes.

Target Gene	Round	Direction	Sequence	Expected Amplicon Size (bp)
16S rRNA	1st	Forward	5′-TACCTGGGTTTGACATGCAC-3′	not determined
		Reverse	5′-CTGCGATTACTAGCGACTCC-3′	
	2nd	Forward	5′-GTGTCGTGAGATGTTGGGTT-3′	200
		Reverse	5′-CCTTTGTACCGGCCATTGTA-3′	
*hsp65*	1st	Forward	5′-CCCCACGATCACCAACGATG-3′	463
		Reverse	5′-CGAGATGTAGCCCTTGTCGAACC-3′	
	2nd	Forward	5′-ACCAACGATGGTGTGTCCAT-3′	439
		Reverse	5′-CTTGTCGAACCGCATACCCT-3′	

**Table 2:** Results of BLAST analysis of partial 16S rRNA and *hsp65* gene sequences.

Target Gene	Tissue	Top BLAST Hit (Accession Number)	Identity (Matches/Length)
16S rRNA	Gill	*M. marinum* strain myco-002 (MG009239.1)	97.3% (146/150)
	Spleen	*M. marinum* strain 2535854 (PP738646.1)	95.7% (202/211)
*hsp65*	Gill	*M. marinum* isolate 275 (MN450812.1)	99.5% (387/389)
	Kidney	*M. marinum* isolate 275 (MN450812.1)	100.0% (312/312)

## Data Availability

The data presented in this study are openly available. The nucleotide sequences obtained in this study were submitted to GenBank (accession numbers: PZ274886, PZ274891, PZ290491, and PZ290492).
